# The Burden of COPD in China and Its Provinces: Findings From the Global Burden of Disease Study 2019

**DOI:** 10.3389/fpubh.2022.859499

**Published:** 2022-06-03

**Authors:** Peng Yin, Jiayuan Wu, Lijun Wang, Chaole Luo, Lihuan Ouyang, Xiantong Tang, Jiangmei Liu, Yunning Liu, Jinlei Qi, Maigeng Zhou, Tianwen Lai

**Affiliations:** ^1^Department of Respiratory and Critical Care Medicine, Affiliated Hospital of Guangdong Medical University, Zhanjiang, China; ^2^National Center for Chronic Noncommunicable Disease Control and Prevention, Chinese Center for Disease Control and Prevention, Beijing, China

**Keywords:** chronic obstructive pulmonary disease, epidemiology, secular trend, China, Global Burden of Disease 2019

## Abstract

In China, chronic obstructive pulmonary disease (COPD) was accounted for a quarter of the global COPD population and has become a large economic burden. However, the comprehensive picture of the COPD burden, which could inform health policy, is not readily available for all of the provinces of China. Here, we aimed to describe the burden of COPD in China, providing an up-to-date and comprehensive analysis at the national and provincial levels, and time trends from 1990 to 2019. Following the methodology framework and general analytical strategies used in the GBD 2019, we analyzed the incidence, prevalence, mortality, disability-adjusted life years (DALYs), years lived with disability (YLDs), and years with life lost (YLLs) attributable to COPD across China and the corresponding time trends from 1990 to 2019, stratified by age and province. In order to quantify the secular trends of the burden of COPD, the estimated annual percentage changes were calculated by the linear regression model of age-standardized rates (ASRs) and calendar years. We also presented the contribution of risk factors to COPD-related mortality and DALYs. The association between COPD burden and socio-demographic index (SDI) were also evaluated. From 1990 to 2019, the incidence and prevalence numbers of COPD increased by 61.2 and 67.8%, respectively, whereas the number of deaths and DALYs owing to COPD decreased. The ASRs of COPD burden, including incidence, prevalence, mortality, DALYs, YLDs, and YLLs continuously decreased from 1990 to 2019. The crude rates of COPD burden dramatically increased with age and reached a peak in the older than 95 years age group. In 2019, the leading risk factor for COPD mortality and DALYs was tobacco use in the whole population, but ambient particulate matter pollution was the most significant risk factor in females. At the provincial level, the ASRs of COPD burden was significantly associated with the SDIs, with the highest ASRs in the western provinces with low SDIs. Collectively, our study indicated that COPD remains an important public health problem in China. Geographically targeted considerations should be developed to enhance COPD health and reduce the COPD burden throughout China and in specific provinces.

## Introduction

Chronic obstructive pulmonary disease (COPD) is one of the leading causes of morbidity and mortality worldwide and results in a huge economic, social and health care burdens (1–4). China is the largest developing country and has the largest number of active smokers (300 million adults) (5). The burden of COPD is expected to dramatically increase in coming decades due to the rapid aging Chinese population. COPD has become the main cause of mortality and disability, resulting in a very large economic burden (6). The most recent Chinese national survey of COPD showed that China accounted for almost 25% of all COPD cases globally. Compared with the survey results 10 years ago, the prevalence of COPD has increased by 67% in aged 40 years or older group in 2012–2015 and has reached epidemic proportions (6).

In China, sociodemographic development, economic shifts, and lifestyle have changed remarkably over the decades. Moreover, with the aging population, continued high prevalence of cigarette smoking, and ambient air pollution, the burden of COPD is anticipated to continue to rise substantially (7, 8). Some studies have been made to assess the burden of COPD in China based on limited localities or small sample sizes, and have no reports summarizing the burden of COPD or a comprehensive compilation of the disability-adjusted life-years (DALYs) and risk exposure for COPD across all provinces of China over time (9–11). Understanding the levels and trends of COPD burden at the finest scale achievable is essential to support evidence-based policy development and targeted prevention and control of COPD in China. We searched PubMed and publicly available reports for articles published up to February, 2021, using the terms “chronic obstructive pulmonary disease,” “COPD,” “burden,” “cause of death,” “incidence,” “prevalence,” “mortality,” “disability-adjusted life years,” “DALY,” “epidemiology,” “China,” “lung diseases,” and “trends.” without language or other publication restrictions. Although previous epidemiologic studies have been made to assess the burden of COPD based on limited localities or small sample sizes, there have been no reports summarizing the burden of COPD or a comprehensive compilation of the time trend and risk exposure for COPD across all provinces of China over time.

The Global Burden of Diseases Study (GBD) 2019 is a systematic scientific effort to provide a comprehensive and updated assessment of the burden of disease across causes of disability and death. To address these data gaps, our study comprehensively assessed COPD burden in all the provinces across the country, and updated the burden estimates and variation trend of COPD in China over three decades from 1990 to 2019. Our study provides important information for planning of public health services through the analysis of COPD, which will be help to identify gaps in COPD health and guiding future policy development of management strategies. Geographically targeted considerations should be developed to enhance COPD health and reduce the COPD burden throughout China and in specific provinces. This research can provide information not only for China's policies, but also for other regions in the world that are experiencing economic growth and shifting the burden of COPD.

## Methods

### Overview

The design and standardized methods of the GBD study have been extensively reported in existing GBD literatures (12–14). The GBD 2019 integrated literature research, monitoring and investigation information, inpatient and outpatient data, medical insurance situation, and other information to evaluate the incidence, prevalence, mortality, and disability adjusted life years of 369 diseases, injuries, and 87 risk factors in 204 countries from 1990 to 2019. The GBD study provides a comprehensive assessment of disease burden by cause, age, sex, year, and location, allowing direct comparisons between different populations, time periods, and regions. The present study focuses on COPD burden, as measured by incidence, prevalence, mortality, and DALYs, in China from 1990 to 2019. A total of 33 provincial administrative units were analyzed in this study, including 31 mainland provinces, municipalities, and autonomous regions, and the Hong Kong and Macao Special Administrative Regions (SAR), all of which are regarded as provinces in the rest of this paper. International Classification of Disease-10 (ICD-10) codes used to represent COPD include J41, J42, J43, J44, and J47, while the corresponding ICD-9 codes are 491–292, and 496. Ethical approval was waived for this study because the GBD data are anonymous and publicly available.

### Estimates of Disease Burden

In summary, the primary data sources used to estimate COPD burden in China were the Disease Surveillance Points System and Cause of Death Reporting System from the Chinese Center for Disease Control and Prevention. The methodological framework of GBD began with mortality estimates. Input data from different sources were procured to produce cause-specific mortality estimates using the Cause of Death Ensemble Model (CODEm). After mapping across different revisions and national variants of the ICD and completing garbage code redistribution, COPD mortality was estimated by province, age, and year. Due to the regional differences of data quality across the country, we applied different under-reporting rate and garbage code redistribution coefficient for a more accurate estimate of COPD mortality. After then, the DisMod-MR 2.1 tool which utilizes Bayesian meta-regression was used to estimate the prevalence, incidence, and years lived with disability (YLDs). Another metric used in the GBD study is the DALYs. The DALYs are the sum of years of life lost (YLLs) and YLDs, which are used to measure the health loss (premature death and disability) due to a specific cause. The YLLs are calculated by multiplying the death number in each age group by the reference life expectancy at the average age of death for those who die in that age group. The YLDs are computed by multiplying the prevalence of each sequela by its disability weight for the corresponding health state plus a comorbidity adjustment. The disability weights proposed by the GBD study are obtained from population-based surveys of countries around the world.

Moreover, other covariates provided by the GBD study included the Socio-demographic index (SDI) and risk exposure. The SDI reflects a location's position on a spectrum of development, which is calculated from the geometric mean of three rescaled components: income per capita, average educational attainment in population aged 15 years or older, and total fertility rates under 25 years. SDI scores ranged from 0 (lowest income, fewest years of schooling, and highest fertility) to 1 (highest income, most years of schooling, and lowest fertility). Risk exposure of COPD mortality and DALYs included six risk factors (smoking, second-hand smoke, household air pollution, ambient particulate matter pollution, ambient ozone pollution, and occupational risks, which include coal dust). Previous reports have clarified the risk factor hierarchy and accompanying definitions of exposure in details (13). The brief description of the six risk factors have been listed in the Supplementary Table 1.

### Statistics

We obtained the annual incident cases, prevalent cases, death number, and DALYs number of COPD in China from 1990 to 2019. Moreover, the burden of COPD was also represented by the age-standardized rates (ASRs) (rates per 100,000), including age-standardized incidence rates (ASIRs), age-standardized prevalence rates (ASPRs), age-standardized mortality rates (ASMRs), and age-standardized DALY rates, at both the national and provincial levels. The ASRs and the 95% uncertainty intervals (UI) were calculated based on the GBD reference population (13). The crude rates and the 95% UIs were also used to compare the COPD burden between different age groups in 2019. Temporal trends in COPD burden from 1990 to 2019 were examined by the estimated annual percentage changes (EAPCs) of ASRs. The EAPC was commonly used to reflect the variation tendency of ASRs over a specified interval. Accordingly, a regression line was fitted to the natural logarithm of the rates: *y* = α + βx + ε, where *y* = ln (ASR) and *x* = calendar year. The EAPC was calculated as 100 × (exp (β) −1), and its 95% UI was obtained from the linear regression model (15). EAPC > 0 and 95% UI > 0 indicated an increasing ASR trend, whereas EAPC estimation <0 and 95% UI did not exceed 0 suggested a decreasing ASR trend. Otherwise, ASRs were regarded as stable over time. To explore the effect of the development status on the COPD burden, we analyzed the strength and direction of the association between the SDIs and the ASIRs, ASMRs, and age-standardized DALY rates at the provincial level through scatter plot and Spearman's rank order correlation analysis. All statistical procedures were performed with the R program (Version 3.5.3, R core team). A *P*-value of <0.05 was considered statistically significant.

## Results

### COPD Burden and Their Trends

The COPD burden, as measured by incidence, prevalence, mortality, and DALYs, and their ASRs in 1990 and 2019, as well as their trends are listed in Table 1. According to the GBD 2019, the number of new cases of COPD increased by 61.2%, from 2.39 million (95% UI: 2.24–2.52) in 1990 to 3.97 million (95% UI: 3.60–4.36) in 2019. The prevalence numbers increased by 67.8%, from 27.75 million (95% UI: 26.16–29.38) in 1990 to 45.17 million (95% UI: 41.13–49.62) in 2019. In contrast, the number of deaths owing to COPD decreased from 1.24 million (95% UI: 0.91–1.40) in 1990 to 1.04 million (95% UI: 0.90–1.27) in 2019. The DALYs number also decreased from 26.12 million (95% UI: 19.59–29.36) in 19.92–19.92 (95% UI: 17.36–23.71) in 2019.

**Table 1:** The number and age-standardized rates of incidence, prevalence, mortality, DALYs, YLDs, and YLLs in 1990 and 2019, and their temporal trends from 1990 to 2019 in China.

	**1990**		**2019**		**EAPC of ASR**
	**Number (Million)**	**ASR (per 100,000)**	**Number (Million)**	**ASR (per 100,000)**	**(1990–2019)**
Incidence	2.39 (2.24–2.52)	288.05 (271.18–302.45)	3.97 (3.60–4.36)	205.89 (188.42–223.62)	−1.31 (−1.40 to −1.21)
Prevalence	27.75 (26.16–29.38)	3300.94 (3104.84–3496.06)	45.17 (41.13–49.62)	2404.42 (2195.92–2636.26)	−1.27 (−1.41 to −1.13)
Mortality	1.24 (0.91–1.40)	217.94 (163.27–242.01)	1.04 (0.90–1.27)	65.20 (55.51–80.09)	−4.53 (−4.75 to −4.31)
DALYs	26.12 (19.59–29.36)	3611.81 (2710.98–4026.49)	19.92 (17.36–23.71)	1102.77 (962.93–1309.04)	−4.44 (−4.63 to −4.26)
YLDs	2.79 (2.31–3.22)	330.33 (274.04–378.75)	4.51 (3.70–5.27)	240.40 (197.20–278.52)	−4.98 (−5.20 to −4.77)
YLLs	23.33 (16.75–26.47)	3281.48 (2393.73–3687.54)	154.11 (13.08–18.94)	862.37 (736.42–1053.64)	−1.25 (−1.43 to −1.06)

*ALYs, disability-adjusted life years; YLDs, years lived with disability; YLLs, years of life lost; ASR, age-standardized rate; EAPC, estimated annual percentage change*.

The ASRs of COPD burden, including incidence, prevalence, mortality, DALYs, YLDs, and YLLs, continuously decreased from 1990 to 2019 (Supplementary Figure 1). The ASIR per 100,000 decreased from 288.05 (95% UI: 271.18–302.45) in 1990 to 205.89 (95% UI: 188.42–223.62) in 2019 by an average of −1.31 (95% UI: −1.40 to −1.21) per year. The same decreased trends were also found in the ASPR per 100,000 from 3300.94 (95% UI: 3104.84–3496.06) in 1990 to 2404.42 (95% UI: 2195.92–2636.26) in 2019 with the EAPC being −1.27 (95% UI: −1.41 to −1.13). The ASMR per 100,000 decreased from 217.94 (95% UI: 163.27–242.01) in 1990 to 65.20 (95% UI: 55.51–80.09) in 2019 with the EAPC being −4.53 (95% UI: −4.75 to −4.31), and the age-standardized DALY rates per 100,000 declined from 3611.81 (95% UI: 2710.98–4026.49) in 1990 to 1102.77 (95% UI: 962.93–1309.04) in 2019 with the EAPC being −4.44 (95% UI: −4.63 to −4.26).

In 2019, the ASIR of COPD for the whole population in China was the same to that of the world. Concerning the prevalence of COPD, the ASRs in China were lower that of the world. However, the ASMR and age-standardized DALY rate of COPD in China were significantly higher than those in the world (Table 2).

**Table 2:** Comparison of age-standardized incidence, prevalence, mortality, DALY, YLD, and YLL rates of COPD in China and in the world, 2019.

**Variable**	**China**	**World**
**(per 100,000)**		
ASIR	205.89 (188.42–223.62)	200.49 (188.63–212.57)
ASPR	2404.42 (2195.92–2636.26)	2638.20 (2492.17–2796.14)
ASMR	65.20 (55.51–80.09)	42.52 (37.63–46.31)
Age-standardized	1102.77 (962.93–1309.04)	926.08 (848.76–997.67)
DALY rate		
Age-standardized	240.40 (197.20–278.52)	245.28 (205.21–276.84)
YLD rate		
Age-standardized	862.37 (736.42–1053.64)	680.80 (606.41–741.65)
YLL rate		

*ASIR, age-standardized incidence rate; ASPR, age-standardized prevalence rate; ASMR, age-standardized mortality rate; DALY, disability-adjusted life years; YLDs, years lived with disability; YLLs, years of life lost; COPD, chronic obstructive pulmonary disease*.

**Figure 1:**
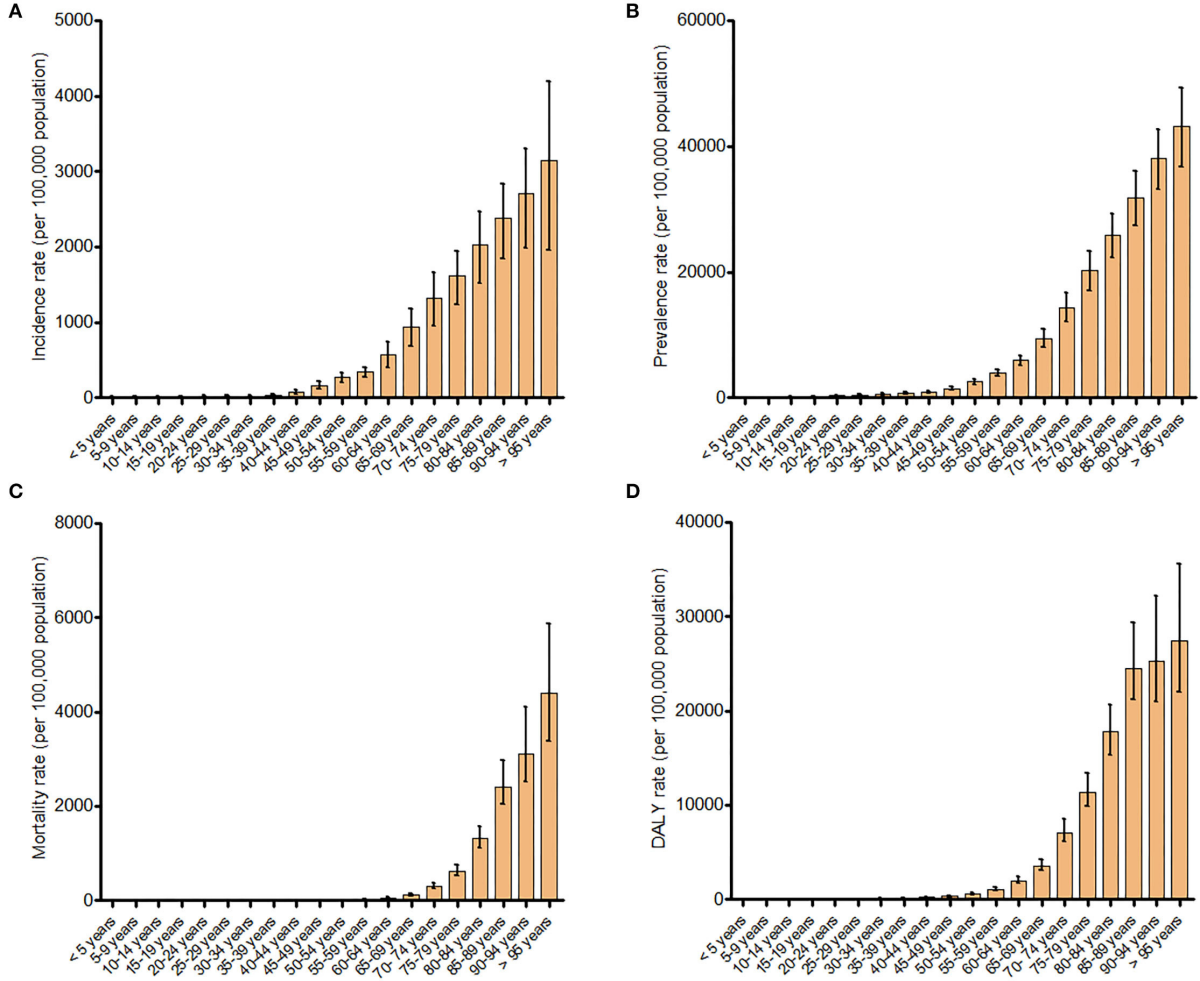
Incidence **(A)**, prevalence **(B)**, mortality **(C)**, and disability-adjusted life years **(D)** rates for chronic obstructive pulmonary disease in different age groups.

The crude incidence rates of COPD per 100,000 dramatically increased with age, and reached a peak at the age group of older than 95 years [3141.48 (95% UI: 1958.32–4189.21)]. The same patterns were seen in terms of prevalence, mortality, and DALYs due to COPD (Figure 1).

In 2019, the COPD-related healthy life loss at the national level was mainly attributed to YLLs, which accounted for 77.36% of DALYs. However, the decrease in ASRs over time was more significant in YLLs [−4.98 (95% UI: −5.20 to −4.77)] than in YLDs [−1.25 (95% UI: −1.43 to −1.06)]. Moreover, the proportions of YLDs in DALYs in 2019 increased across the country and in 33 provinces relative to those in 1990. The provinces with high SDI, including Tianjin, Hong Kong, Beijing, and Liaoning, had a higher proportion of YLDs in DALYs than other provinces (Supplementary Figure 2).

### Risk Factors for COPD Burden

**Figure 2:**
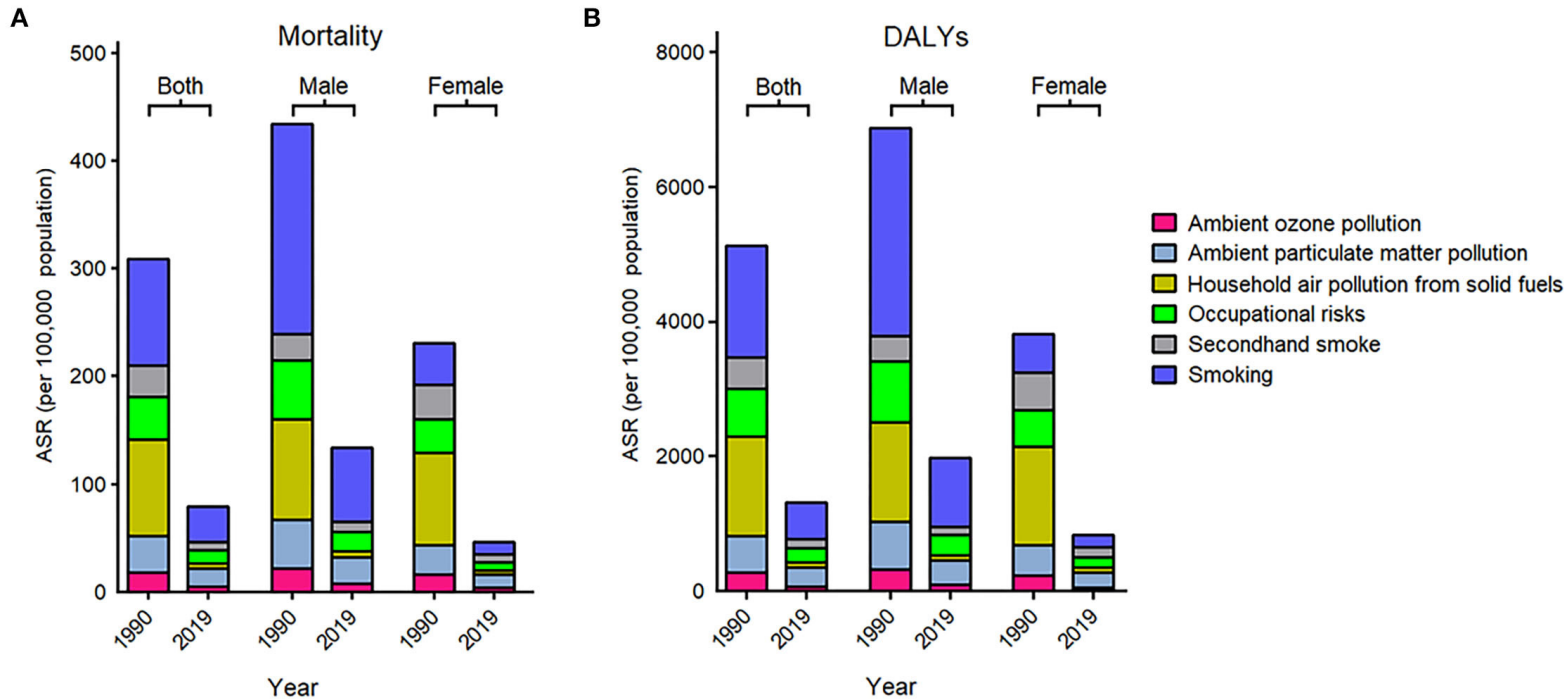
Age-standardized rates of mortality **(A)** and disability-adjusted life years **(B)** for chronic obstructive pulmonary disease by risk factor and sex in 1990 and 2019.

Overall, the ASMRs and age-standardized DALYs rates of COPD attributable to different risk factors in 2019 significantly declined as compared to those in 1990 (Figure 2). In 2019, smoking was the most common risk factor for COPD burden in both sexes across the country, contributed to an ASMR per 100,000 of 33.02 (95% UI: 27.53–39.51) and 50.70% (95% UI: 45.66–54.88) of COPD-related death, as well as an age-standardized DALYs rate per 100,000 of 543.33 (95% UI: 462.20–645.36) and 49.27% (95% UI: 44.80–53.11) of COPD-related DALYs. Similarly, smoking also represented the most prominent risk factor for COPD burden in men, responsible for an ASMR per 100,000 of 68.89 (95% UI: 57.35–81.89) and 73.74% (95% UI: 70.64–77.06) of COPD-related death, as well as an age-standardized DALY rate per 100,000 of 1023.13 (95% UI: 857.00–1207.79) and 73.84% (95% UI: 71.11–76.74) of COPD-related DALYs. However, among women, ambient particulate matter pollution were the leading risk factors for COPD burden, accounting for an ASMR per 100,000 of 12.08 (95% UI: 8.66–17.42) and 24.93% (95% UI: 20.25–30.38) of COPD-related mortality, as well as an age-standardized DALY rate per 100,000 of 224.70 (95% UI: 169.87–308.45) and 24.76% (95% UI: 20.10–30.14) of COPD-related DALYs.

### COPD Burden and Their Trends at the Provincial Level

**Figure 3:**
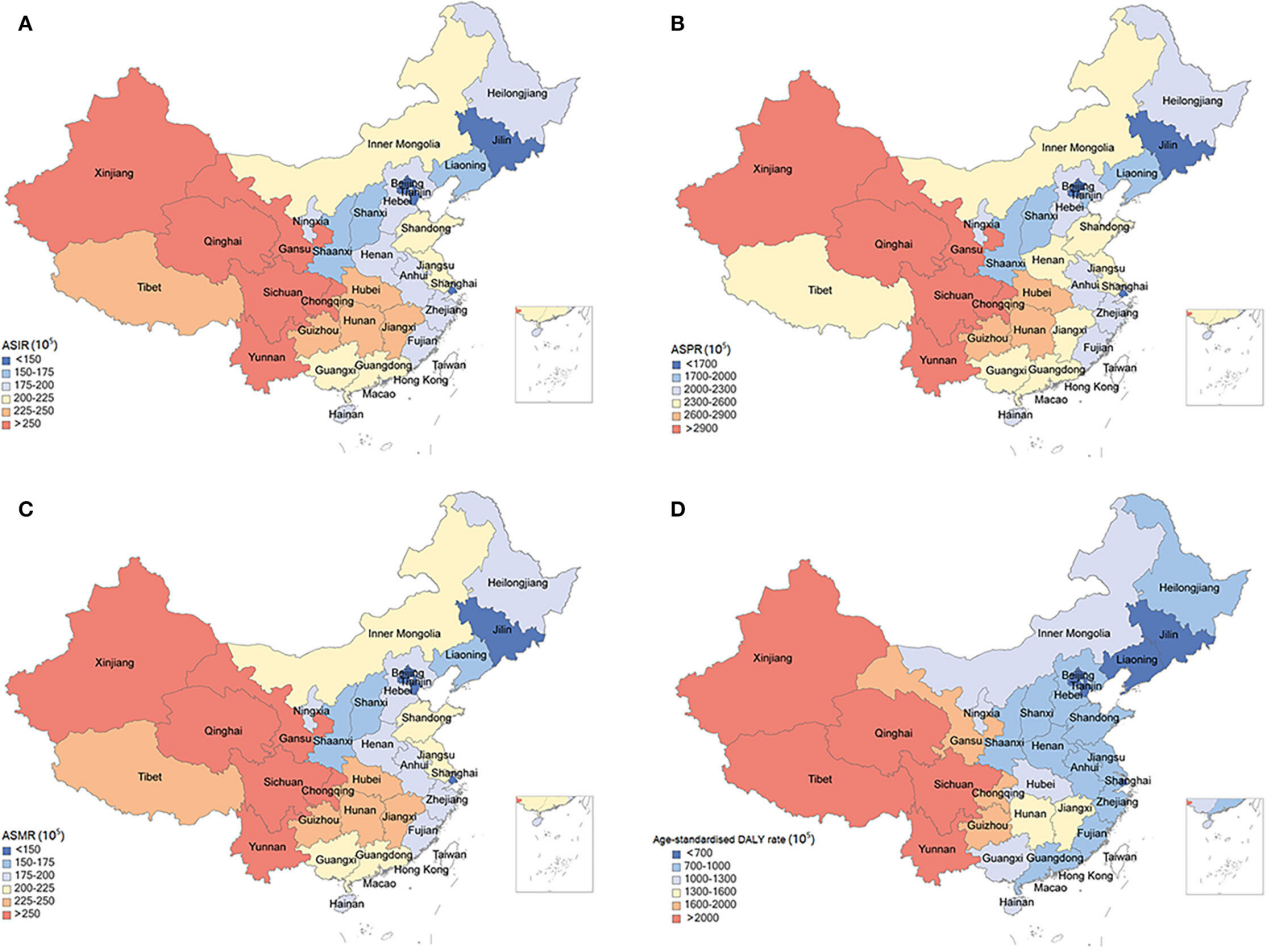
Age-standardized rates of incidence **(A)**, prevalence **(B)**, mortality **(C)**, and disability-adjusted life years **(D)** for chronic obstructive pulmonary disease by location in China, 2019.

The COPD burden of the 33 provinces in 2019 were shown in Figure 3. Totally, higher ASIR, ASPR, ASMR, and age-standardized DALY rates of COPD were seen in the Western provinces in China. The highest ASIR was observed in Sichuan [266.51 (95% UI: 245.44–286.26) per 100,000], which showed 2.12-fold differences compared with the lowest ASIR in Beijing [125.68 (95% UI: 112.22–140.46) per 100,000) (Supplementary Table 2). Similarly, the highest ASPR was also observed in Sichuan [3263.63 (95% UI: 3010.40–3538.41) per 100,000], which was 1.27 times higher than the lowest ASPR in Beijing [1435.59 (95% UI: 1273.79–1609.32) per 100,000) (Supplementary Table 3). The highest ASMR was detected in Tibet [181.44 (95% UI: 146.11–215.83) per 100,000], which was 8.01 times higher than the lowest ASMR in Hong Kong [20.14 (95% UI: 13.69–38.47) per 100,000] (Supplementary Table 4). Moreover, the highest age-standardized DALY rate was observed in Tibet [2943.41 (95% UI: 2397.16–3479.53) per 100,000], which was 6.88-fold differences compared with the lowest age-standardized DALY rate in Beijing [427.65 (95% UI: 345.77–627.78) per 100,000] (Supplementary Table 5).

**Figure 4:**
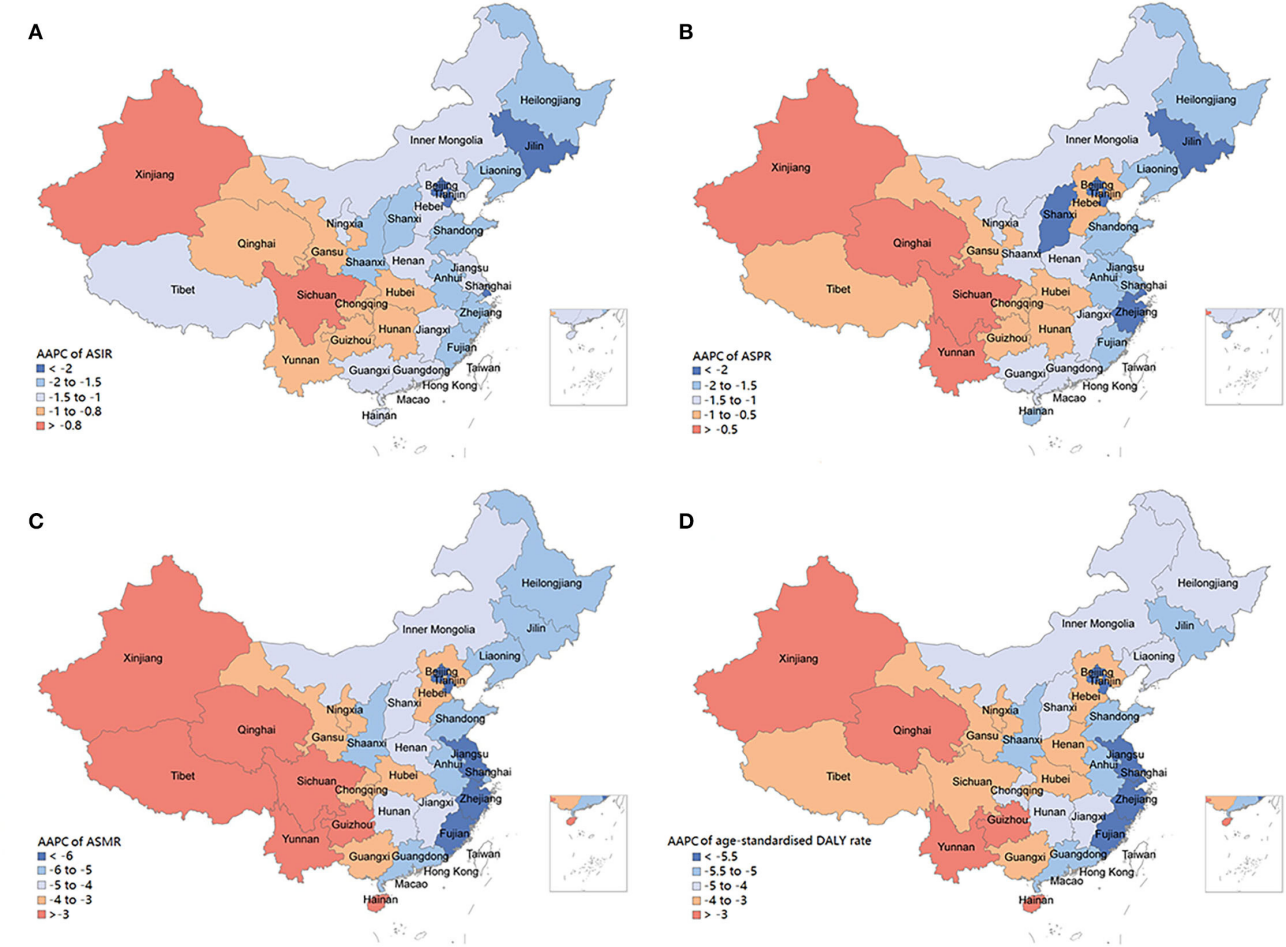
Average annual percentage changes of incidence **(A)**, prevalence **(B)**, mortality **(C)**, and disability-adjusted life years **(D)** for chronic obstructive pulmonary disease by location in China, 1990-2019.

Overall, the COPD burden declined in all the 33 provinces during the observed period, and the most significant declines were found in the Eastern provinces rather than the Western provinces (Figure 4). Beijing had the most significant reduction of ASIRs [−2.63 (95% UI: −2.80 to −2.46)] and ASPRs [−2.78 (95% UI: −2.99 to −2.57)], whereas Sichuan had the lowest change in ASIRs [−0.54 (95% UI: −0.61 to −0.46)] and ASPRs [−0.29 (95% UI: −0.40 to −0.18)] (Supplementary Tables 2, 3). The largest decline of ASMRs was observed in Tianjin [−6.56 (95% UI: −6.88 to −6.23)], whereas the smallest decrease in Hainan [−2.18 (95% UI: −2.42 to −1.94)] (Supplementary Table 4). In addition, Zhejiang showed the top decrease in age-standardized DALY rates [−6.18 (95% UI: −6.51 to −5.86)], whereas Qinghai showed the minimum reduction [−2.62 (95% UI: −2.74 to −2.49)] (Supplementary Table 5).

From 1990 to 2019, the age-standardized YLD and YLL rates of COPD also showed a continuous decreasing trend at provincial level of China, of which the results are shown in Supplementary Tables 6, 7, respectively.

### Correlation Between SDIs and Estimates of Incidence, Mortality, and DALYs

**Figure 5:**
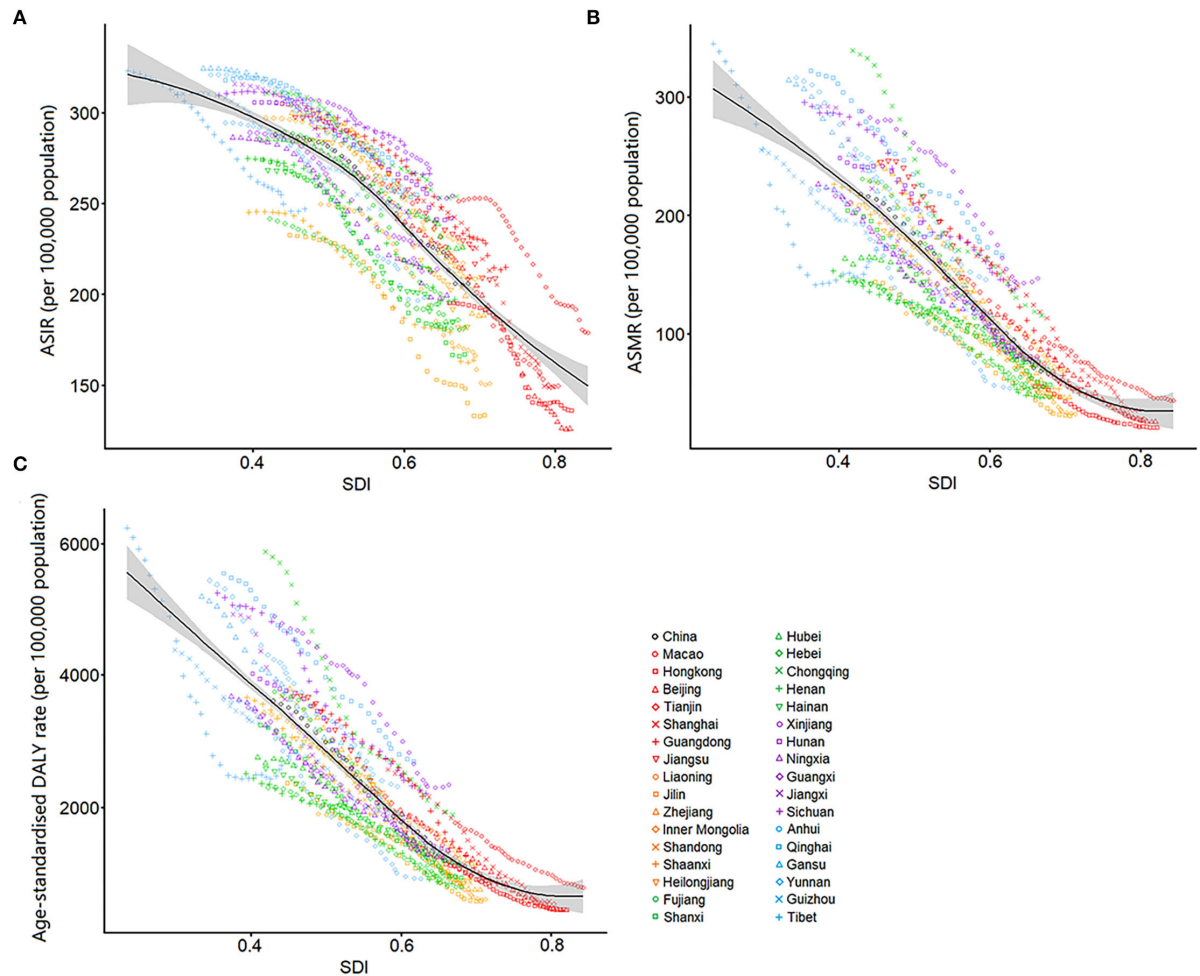
Age-standardized rates of incidence **(A)**, mortality **(B)**, and disability-adjusted life years **(C)** for chronic obstructive pulmonary disease by Socio-demographic Index in China, 1990–2019. The black line represents expected values of age-standardized rates for each value of SDI.

Associations between SDIs and COPD burden estimates over time for each province are shown in Figure 5. The SDIs continuously increased in all provinces during 1990 to 2019. The ASIRS, ASMRs, and age-standardized DALYs rates across the country and in 33 provinces generally decreased as their SDI has increased. The SDIs had a strongly negative correlation with ASIRs (*r* = −0.83, *P* < 0.001), ASMRs (*r* = −0.86, *P* < 0.001), and age-standardized DALY rates (*r* = −0.85, *P* < 0.001).

## Discussion

To the best of our knowledge, this study provided the most comprehensive and latest assessment of the burden of COPD in China. The standardized methods used in the GBD study made it possible to compare the COPD burden in China with that at the global level. From 1990 to 2019, the ASRs of COPD burden, especially mortality and DALYs, have been dramatically decreased. This decline was mainly attributed to the rapid socioeconomic development in China during the past three decades as a result of better nutrition supply, living conditions, education, and health care, which can effectively reduce the risk of chronic diseases. However, the absolute number of COPD incidence and prevalence have an increasing trend with a nearly two-thirds increment in 2019 than in 1990, which was contrary to the variation in the corresponding ASRs. According to the GBD estimates, China represented 24% of all new diagnosis, 21% of all prevalent cases, 32% of all deaths, and 27% of all DALYs from COPD across the world in 2019 (12). Why does China have such a heavy burden of COPD? One major reason for the rise of COPD is population aging. Whereas, there were 13 million Chinese residents older than 70 years in 1970, it has climbed to over 80 million now and will be 215 million by 2050 (16). Other potential reason is cigarette smoking. According to a cross-section study recruited more than 60,000 adults aged over 40 years across all major provinces in China, it astonishingly found that 58% of the male participants were smokers, whereas only 4% of female participants have the experience of smoking (17). As COPD contributes to an immense economic burden, as well as a considerable impairment on physiological function and quality of life of patients, our study highlighted that COPD continues to cause a heavy burden on patients, the health-care system, and society in China that call for aggressive preventive measures and management strategies to defeat COPD in China.

Despite decrease in age-standardized rates of COPD burden, China still has substantially higher COPD burden in mortality and DALYs than the global average. This high rate of health loss from COPD might be the result of its relatively late diagnosis and management (18). Evidence suggests that COPD is underdiagnosed in China partly due to diagnosis is often based on symptoms rather than spirometry (19). Asymptomatic patients are often late in seeking care and do not ask for medical care until the symptoms interfere with their daily life. Notably, asymptomatic patients who are in urgent need for early diagnosis account for a considerable proportion (35.1%) of all COPD patients, which represents a major challenge in China (20). Physicians should be trained and community health-care centers should be provided with spirometers to help early diagnose COPD patients in high-risk population, such as those with chronic sputum production or dyspnea, or heavy smokers.

In view of the relationship between the COPD burden and age, our data confirms that COPD is a progressive disease. The COPD burden dramatically increased with age, and reached a peak at the age group of older than 95 years. This increase was most prominent in middle aged and older populations. The primary drivers of this increase were longer life expectancy in China and continuous exposure to risk factors, such as indoor and outdoor pollution. However, it was important to note that COPD can also be present early in life. Wang et al. showed that the prevalence of COPD was 2.1% among the young adults aged 20–39 years in China (6). Therefore, young populations should be included in COPD screening programs.

Despite the decrease of premature death caused by COPD, YLD increased significantly, and the proportion of YLD in DALY increased simultaneously, indicating that the burden of disability caused by COPD is on the rise, which is a lot to do with the limited treatment of advanced disease. Currently, the detection rate of lung function in China is very low (21). Many patients with obvious symptoms of cough and sputum do not receive regular pulmonary function examination, resulting in the delay diagnosis of the disease. When many patients are diagnosed, their respiratory obstruction has reached the moderate to severe level (22). Most patients continue to develop limited motor function, resulting in disability and a serious burden on their families and society. Therefore, the level of early detection should be improved to avoid the disabling disease burden caused by the development of the disease from early to late stage.

The burden of COPD shows substantial heterogeneity in China at the province level, with higher ASRs were observed in the less-developed western provinces, such as Sichuan, Tibet, and Gansu. The variation in COPD burden over time showed the same trend among these provinces, with the least decline in the western provinces. A strongly negative association between COPD burden and socioeconomic development can partly explain this inequality of COPD burden at the provincial level. The low socioeconomic development is associated with poor health awareness, inadequate social support, lack of medical care, poor living environment, and increased exposure to some risk factors, such as smoking and biomass fuels. These factors interact with each other to some extent and directly or indirectly lead to the development of COPD. Even in a well-developed country with an advanced health care system, low socioeconomic status, representing as short length of school education, is associated with a poorer prognosis of COPD (23). Indoor air pollution from the burning of coal and biomass fuels is a major cause of COPD in the developing countries. The use of solid fuels is inversely associated with socioeconomic development, because it is very likely that the home of poor people are places where lung injury caused by smoke from solid fuel. Since the 1990s, China has done a lot in the promotion of clean energy (24). Most developed urban areas have basically used cleaner and more effective electricity or natural gas for cooking and heating. However, in the less developed regions, especially in the western China, the penetration rate of clean energy and indoor ventilation equipment is still low, whereas the utilization of solid fuel is still high, leading to a high exposure to this risk factor of COPD. Studies have showed that the occupational exposure to dust and harmful gas is much higher in the western regions than in the central and eastern regions, which is estimated to be related to the higher distribution of mineral resources in the western China (25). Moreover, the cold climate and low average air humidity in the western provinces, such as Tibet, Qinghai, and Gansu, is strongly associated with the high COPD burden (26). In addition, the low lung function detection rate and treatment rate due to socioeconomic underdevelopment further exacerbate the burden of COPD in these regions. A national survey across seven provinces found that only 30% of COPD patients had been diagnosed with COPD-related respiratory disease, 2.4% had received lung function test, and 7.9% had underwent regular medication in the less developed regions of China (27). Therefore, COPD patients in China still have a large amount of unmet needs in terms of health services.

In 2019, the COPD-related mortality and DALYs attributable to all risk factors have been significantly reduced as compared to those in 1990 for the whole population, but smoking is still the most important risk factor. Notably, ambient particular matter pollution has become the most and second important risk factors in 2019, whereas it only ranked fifth and fourth among all risk factors in 1990 for women and men, respectively. With rapid industrialization, air pollution has become the biggest environmental challenge to public health in China. Growing body of evidence have revealed that increased daily mean PM2.5 and PM10 concentrations were significantly associated with increased COPD prevalence and declined respiratory function (28, 29). However, the majority of evidence on the effects of air pollution on COPD in China are based on cross-sectional data. So far, there has been no long-term cohort study of respiratory health and air pollution in China. In Europe, studies have shown that reducing exposure to airborne particulate matters appears to attenuate the decline in lung function caused by the exposure to PM (30). For the past decade, Chinese government has showed its ambition and done a lot in the management of air pollution, which partly contributed to the reduction of COPD burden, and might serve as an example for the developing countries (31).

Compared with previous studies on COPD burden in China, the present study showed the following strengths. First, our study analyzed the COPD burden, as measured by incidence, prevalence, mortality, and DALYs, in the Chinese population to provide more comprehensive information and explicit guidance for the future policy development of management strategies. Second, this study for the first time revealed the trend of COPD burden over time, which fill the gap in the long-term trend of the COPD burden in China. Third, we firstly explored the significant association between COPD burden and socioeconomic development, that can supply a scientific basis for COPD controlling and health facilities planning. However, several limitations should be acknowledged which are subjected to all the limitations of the GBD studies. First, the GBD 2019 estimated the models of COPD mainly through collecting as much published and unpublished data as possible at the national and provincial levels. The absence of relevant data in some provinces, especially in western China, might have led to measurement bias in the model estimates. Second, although the quality of ICD coding in death certificates in China has improved during the study period, there were substantial differences between provinces. Particularly, long-term trends in low SDI provinces with high mortality, such as Tibet, may be influenced by the redistribution of garbage codes. Third, because most COPD patients were identified by spirometry, underdeveloped regions with a lack of spirometric evaluation might have a high rate of misdiagnosis and underdiagnosis. Fourth, although the uncertainty was propagated in each step of modeling strategy in the GBD studies, some uncertainties might have not been fully captured in some regions without sufficient data.

## Conclusion

In summary, although the ASRs of COPD burden in China substantial declines at the national and provincial levels from 1990 to 2019, absolute numbers of COPD incidence and prevalence are still on rise, indicating that COPD remains an important public health problem in China and high health care costs are needed in the future due to rapidly aging Chinese population. Policymakers should remain aware that the number of incident and prevalent cases represents the burden of COPD that the country's health systems must manage. COPD burden in China is unevenly distributed among age and provinces. Geographically targeted considerations should be developed to enhance COPD health and reduce the COPD burden throughout China and in specific provinces. Moreover, the pattern of risk factors of COPD has changed over time. Our results could help government to understand the COPD burden and develop customized health and medical strategies to reduce the burden of COPD.

## Data Availability Statement

The original contributions presented in the study are included in the article/Supplementary Material, further inquiries can be directed to the corresponding author/s.

## Ethics Statement

The studies involving human participants were reviewed and approved by the Ethics of Research Committee of the Affiliated Hospital of Guangdong Medical University. The patients/participants provided their written informed consent to participate in this study.

## Author Contributions

MZ, TL, PY, and JW conceived the study conception and design and were responsible for analysis and interpretation. TL, JW, and PY drafted the manuscript based on comments from all other authors. CL, JL, YL, XT, and JQ were responsible for China using Global Burden of Diseases, Injuries, and Risk Factors Study 2019 methods. LW, CL, and LO contributed to the analysis and reviewed the manuscript. All authors contributed to the article and approved the submitted version.

## Funding

This work was supported by the National Natural Science Foundation of China (Nos. 81873404 and 82170030), the Guangdong Basic and Applied Basic Research Foundation (Nos. 2020B1515020004 and 2020A1515110804), Project of Young Innovative Talents in Colleges and Universities in Guangdong Province (No. 2018KQNCX095), Affiliated Hospital of Guangdong Medical University Clinical Research Program (LCYJ2019B011).

## Conflict of Interest

The authors declare that the research was conducted in the absence of any commercial or financial relationships that could be construed as a potential conflict of interest.

## Publisher's Note

All claims expressed in this article are solely those of the authors and do not necessarily represent those of their affiliated organizations, or those of the publisher, the editors and the reviewers. Any product that may be evaluated in this article, or claim that may be made by its manufacturer, is not guaranteed or endorsed by the publisher.

## Abbreviations

ASR: age-standardized rate
COPD: Chronic obstructive pulmonary disease
DALYs: disability-adjusted life years
EAPC: estimated annual percentage change
GBD: Global Burden of Diseases Study
YLDs: years lived with disability
YLLs: years of life lost.
