# Supplementary material for: The Burden of COPD in China and Its Provinces: Findings From the Global Burden of Disease Study 2019
Source: Front Public Health. 2022 Jun 3;10:859499. doi: 10.3389/fpubh.2022.859499 (PMC9215345; doi:10.3389/fpubh.2022.859499)
Supplement: Supplementary file 3 [file Data_Sheet_1.zip › Table 1.DOCX]

**Supplementary Table 1. The brief definition of the six risk factors of COPD.**

| **Risk factor** | **Brief definition** |
| --- | --- |
| Ambient ozone pollution | Exposure to ozone in ambient air (the highest seasonal [six-month] average of eight-hour daily maximum ozone concentrations, in parts per billion [ppb]). |
| Ambient particulate matter pollution | Exposure to ambient particulate matter pollution is defined as the population-weighted annual average mass concentration of particles with an aerodynamic diameter less than 2.5 micrometers (PM2.5) in a cubic meter of air. This measurement is reported in µg/m3. |
| Household air pollution | Exposure to household air pollution from solid fuels (HAP) is estimated from both the proportion of individuals using solid cooking fuels and the level of PM2.5 air pollution exposure for these individuals.  Solid fuels include coal, wood, charcoal, dung, and agricultural residues. |
| Smoking | Smoking in the GBD 2019 includes current smokers and former smokers. Current smokers are defined as individuals who currently use any smoked tobacco product on a daily or occasional basis and former smokers are defined as individuals who quit using all smoked tobacco products for at least six months, where possible, or according to the definition used by the survey. |
| Second-hand smoke | Secondhand smoke exposure is defined as current exposure to secondhand tobacco smoke at home, at work, or in other public places. |
| Occupational risks | Proportion of the population occupationally exposed to asbestos, asthmagens, carcinogens, ergonomic factors, injuries, noise, and particles. |
