# Supplementary material for: The Burden of COPD in China and Its Provinces: Findings From the Global Burden of Disease Study 2019
Source: Front Public Health. 2022 Jun 3;10:859499. doi: 10.3389/fpubh.2022.859499 (PMC9215345; doi:10.3389/fpubh.2022.859499)
Supplement: Supplementary file 3 [file Data_Sheet_1.zip › Table 2.DOCX]

**Supplementary Table 2. The age-standardized incidence rates of COPD in 1990 and 2019, and their temporal trends from 1990 to 2019 at provincial level of China.**

| Province | ASR in 1990 (per 100,000) | | ASR in 2019 (per 100,000) | | EAPC (1990-2019) | |
| --- | --- | --- | --- | --- | --- | --- |
| Anhui | 294.59 (276.38 – 310.20) | 197.95 (178.87 – 219.66) | | -1.58 (-1.71 – -1.44) | |  |
| Beijing | 241.55 (220.71 – 262.53) | 125.68 (112.22 – 140.46) | | -2.63 (-2.80 – -2.46) | |  |
| Chongqing | 310.72 (292.79 – 324.00) | 253.82 (234.39 – 273.77) | | -0.81 (-0.86 – -0.76) | |  |
| Fujian | 289.85 (272.35 – 305.78) | 181.51 (163.00 – 201.16) | | -1.85 (-1.98 – -1.72) | |  |
| Gansu | 324.30 (310.29 – 335.23) | 257.33 (238.92 – 276.75) | | -0.88 (-0.96 – -0.81) | |  |
| Guangdong | 291.63 (272.97 – 308.34) | 214.84 (194.61 – 235.00) | | -1.21 (-1.29 – -1.12) | |  |
| Guangxi | 289.12 (268.14 – 307.75) | 213.97 (194.62 – 235.16) | | -1.17 (-1.25 – -1.10) | |  |
| Guizhou | 310.09 (289.85 – 326.81) | 248.74 (227.08 – 269.81) | | -0.85 (-0.93 – -0.77) | |  |
| Hainan | 268.37 (246.05 – 290.89) | 184.94 (166.56 – 205.88) | | -1.48 (-1.58 – -1.38) | |  |
| Hebei | 241.69 (219.31 – 264.71) | 185.78 (166.38 – 207.11) | | -1.03 (-1.10 – -0.96) | |  |
| Heilongjiang | 284.13 (266.20 – 299.87) | 181.96 (163.79 – 200.37) | | -1.73 (-1.91 – -1.56) | |  |
| Henan | 274.78 (254.15 – 294.97) | 197.16 (177.52 – 219.45) | | -1.30 (-1.42 – -1.19) | |  |
| Hong Kong * | 195.17 (177.16 – 215.85) | 135.72 (121.04 – 151.84) | | -1.59 (-1.76 – -1.42) | |  |
| Hubei | 285.39 (265.41 – 304.09) | 225.77 (206.38 – 247.37) | | -0.90 (-0.97 – -0.83) | |  |
| Hunan | 305.68 (290.01 – 318.80) | 241.96 (221.66 – 262.92) | | -0.89 (-0.96 – -0.82) | |  |
| Inner Mongolia | 297.17 (279.06 – 313.59) | 209.42 (190.04 – 229.78) | | -1.36 (-1.48 – -1.25) | |  |
| Jiangsu | 297.61 (281.24 – 313.55) | 208.83 (189.54 – 228.54) | | -1.36 (-1.46 – -1.26) | |  |
| Jiangxi | 315.91 (300.14 – 328.31) | 228.30 (208.50 – 248.33) | | -1.27 (-1.35 – -1.18) | |  |
| Jilin | 232.54 (213.21 – 254.00) | 133.41 (119.16 – 148.89) | | -2.25 (-2.46 – -2.04) | |  |
| Liaoning | 249.71 (230.31 – 269.51) | 150.58 (134.87 – 169.08) | | -2.00 (-2.16 – -1.83) | |  |
| Macao * | 251.20 (229.19 – 272.42) | 178.87 (160.92 – 198.60) | | -1.39 (-1.50 – -1.27) | |  |
| Ningxia | 286.30 (265.69 – 306.02) | 199.32 (178.70 – 220.20) | | -1.44 (-1.55 – -1.32) | |  |
| Qinghai | 318.04 (299.95 – 332.46) | 254.70 (232.23 – 276.22) | | -0.84 (-0.94 – -0.73) | |  |
| Shaanxi | 245.12 (223.83 – 267.30) | 163.65 (147.04 – 182.64) | | -1.61 (-1.74 – -1.48) | |  |
| Shandong | 304.80 (288.96 – 317.85) | 203.25 (185.14 – 223.40) | | -1.58 (-1.71 – -1.46) | |  |
| Shanghai | 260.07 (238.11 – 280.28) | 148.84 (132.99 – 166.65) | | -2.23 (-2.41 – -2.04) | |  |
| Shanxi | 274.20 (251.76 – 295.66) | 166.85 (149.56 – 187.10) | | -1.98 (-2.11 – -1.84) | |  |
| Sichuan | 309.70 (292.15 – 323.94) | 266.51 (245.44 – 286.26) | | -0.54 (-0.61 – -0.46) | |  |
| Tianjin | 265.46 (244.27 – 286.39) | 149.36 (133.52 – 167.54) | | -2.35 (-2.50 – -2.19) | |  |
| Tibet | 323.05 (305.30 – 338.14) | 246.93 (224.94 – 270.30) | | -1.09 (-1.15 – -1.03) | |  |
| Xinjiang | 305.62 (286.47 – 322.33) | 254.15 (233.03 – 273.99) | | -0.69 (-0.78 – -0.61) | |  |
| Yunnan | 320.99 (304.08 – 333.33) | 259.54 (238.15 – 278.94) | | -0.80 (-0.87 – -0.73) | |  |
| Zhejiang | 299.83 (284.84 – 312.30) | 188.31 (170.18 – 208.70) | | -1.79 (-1.95 – -1.63) | |  |

* Special Administrative Region of China. ASR, age-standardized rate; EAPC, estimated annual percentage change.
