# Supplementary material for: The Burden of COPD in China and Its Provinces: Findings From the Global Burden of Disease Study 2019
Source: Front Public Health. 2022 Jun 3;10:859499. doi: 10.3389/fpubh.2022.859499 (PMC9215345; doi:10.3389/fpubh.2022.859499)
Supplement: Supplementary file 3 [file Data_Sheet_1.zip › Table 3.DOCX]

**Supplementary Table 3. The age-standardized prevalence rates of COPD in 1990 and 2019, and their temporal trends from 1990 to 2019 at provincial level of China.**

| Province | ASR in1990 (per 100,000) | ASR in 2019 (per 100,000) | EAPC (1990-2019) |
| --- | --- | --- | --- |
| Anhui | 3364.12 (3141.88 – 3601.04) | 2269.52 (2038.43 – 2531.91) | -1.62 (-1.80 – -1.44) |
| Beijing | 2866.67 (2624.01 – 3136.63) | 1435.59 (1273.79 – 1609.32) | -2.78 (-2.99 – -2.57) |
| Chongqing | 3637.19 (3434.31 – 3789.29) | 3189.15 (2935.88 – 3442.38) | -0.58 (-0.70 – -0.46) |
| Fujian | 3349.16 (3124.87 – 3580.25) | 2058.34 (1829.02 – 2295.21) | -1.96 (-2.13 – -1.79) |
| Gansu | 3413.50 (3231.11 – 3549.53) | 2982.12 (2748.59 – 3244.82) | -0.57 (-0.68 – -0.45) |
| Guangdong | 3397.92 (3157.88 – 3652.80) | 2484.93 (2239.01 – 2737.50) | -1.24 (-1.37 – -1.11) |
| Guangxi | 3222.40 (2990.86 – 3463.70) | 2423.28 (2194.09 – 2682.77) | -1.14 (-1.25 – -1.03) |
| Guizhou | 3264.32 (3052.77 – 3466.25) | 2866.60 (2613.42 – 3138.27) | -0.58 (-0.68 – -0.47) |
| Hainan | 3044.35 (2789.33 – 3324.16) | 2068.21 (1860.71 – 2310.73) | -1.56 (-1.68 – -1.44) |
| Hebei | 2720.56 (2475.47 – 3002.28) | 2131.51 (1907.22 – 2395.27) | -0.97 (-1.04 – -0.90) |
| Heilongjiang | 3360.19 (3141.74 – 3569.41) | 2184.91 (1972.67 – 2425.22) | -1.70 (-1.92 – -1.48) |
| Henan | 3133.87 (2896.69 – 3403.64) | 2324.65 (2090.32 – 2588.70) | -1.19 (-1.34 – -1.05) |
| Hong Kong * | 2547.11 (2323.11 – 2795.70) | 1673.18 (1498.09 – 1865.74) | -1.85 (-2.04 – -1.65) |
| Hubei | 3264.65 (3012.76 – 3519.79) | 2608.48 (2368.88 – 2888.19) | -0.92 (-1.03 – -0.80) |
| Hunan | 3396.38 (3205.44 – 3567.60) | 2830.96 (2581.81 – 3103.01) | -0.73 (-0.86 – -0.59) |
| Inner Mongolia | 3373.65 (3165.58 – 3587.33) | 2434.30 (2206.68 – 2687.43) | -1.32 (-1.50 – -1.13) |
| Jiangsu | 3533.67 (3301.81 – 3781.82) | 2390.42 (2157.38 – 2641.65) | -1.54 (-1.69 – -1.38) |
| Jiangxi | 3436.19 (3227.40 – 3615.53) | 2581.93 (2343.79 – 2830.20) | -1.17 (-1.32 – -1.02) |
| Jilin | 2690.88 (2473.45 – 2929.26) | 1593.80 (1422.77 – 1775.51) | -2.16 (-2.40 – -1.91) |
| Liaoning | 2922.79 (2679.22 – 3175.71) | 1800.64 (1611.97 – 2023.44) | -1.91 (-2.09 – -1.73) |
| Macao * | 3111.27 (2858.98 – 3389.30) | 2089.75 (1877.71 – 2335.80) | -1.68 (-1.83 – -1.52) |
| Ningxia | 3141.66 (2911.95 – 3389.75) | 2294.92 (2067.64 – 2539.67) | -1.32 (-1.48 – -1.16) |
| Qinghai | 3276.20 (3093.75 – 3436.59) | 2909.90 (2659.26 – 3168.68) | -0.48 (-0.61 – -0.34) |
| Shaanxi | 2708.80 (2478.68 – 2970.98) | 1922.14 (1728.24 – 2143.65) | -1.39 (-1.51 – -1.27) |
| Shandong | 3636.18 (3429.70 – 3826.18) | 2449.82 (2223.15 – 2699.49) | -1.57 (-1.75 – -1.39) |
| Shanghai | 3176.98 (2917.02 – 3464.51) | 1598.08 (1412.71 – 1793.73) | -2.76 (-3.03 – -2.49) |
| Shanxi | 3113.68 (2854.07 – 3389.17) | 1874.98 (1670.44 – 2103.73) | -2.05 (-2.23 – -1.86) |
| Sichuan | 3549.29 (3350.62 – 3715.67) | 3263.64 (3010.40 – 3538.41) | -0.29 (-0.40 – -0.18) |
| Tianjin | 3172.27 (2906.83 – 3448.20) | 1771.34 (1581.91 – 1976.49) | -2.40 (-2.59 – -2.21) |
| Tibet | 3204.90 (3021.90 – 3362.47) | 2553.71 (2334.70 – 2813.33) | -0.97 (-1.04 – -0.90) |
| Xinjiang | 3210.36 (3014.61 – 3409.37) | 2975.65 (2747.36 – 3211.43) | -0.32 (-0.44 – -0.20) |
| Yunnan | 3335.48 (3138.63 – 3492.39) | 2991.59 (2740.14 – 3257.65) | -0.45 (-0.55 – -0.35) |
| Zhejiang | 3554.93 (3365.04 – 3737.31) | 2107.38 (1876.47 – 2349.49) | -2.07 (-2.32 – -1.83) |

* Special Administrative Region of China. ASR, age-standardized rate; EAPC, estimated annual percentage change.
