# Supplementary material for: The Burden of COPD in China and Its Provinces: Findings From the Global Burden of Disease Study 2019
Source: Front Public Health. 2022 Jun 3;10:859499. doi: 10.3389/fpubh.2022.859499 (PMC9215345; doi:10.3389/fpubh.2022.859499)
Supplement: Supplementary file 3 [file Data_Sheet_1.zip › Table 4.DOCX]

**Supplementary Table 4. The age-standardized mortality rates of COPD in 1990 and 2019, and their temporal trends from 1990 to 2019 at provincial level of China.**

| Province | ASR in 1990 (per 100,000) | ASR in 2019 (per 100,000) | EAPC (1990-2019) |
| --- | --- | --- | --- |
| Anhui | 222.36 (167.38 – 257.16) | 53.60 (42.13 – 77.92) | -5.33 (-5.60 – -5.05) |
| Beijing | 118.43 (100.63 – 133.68) | 24.99 (18.91 – 40.61) | -6.21 (-6.54 – -5.89) |
| Chongqing | 339.26 (185.77 – 401.73) | 115.18 (86.48 – 137.95) | -3.85 (-3.96 – -3.74) |
| Fujian | 230.50 (161.19 – 266.03) | 46.15 (36.88 – 64.96) | -6.06 (-6.42 – -5.70) |
| Gansu | 313.97 (218.47 – 360.12) | 115.34 (96.48 – 135.53) | -3.48 (-3.66 – -3.29) |
| Guangdong | 219.02 (153.88 – 251.42) | 47.62 (39.54 – 58.06) | -5.71 (-6.21 – -5.22) |
| Guangxi | 216.42 (161.86 – 247.82) | 79.77 (64.34 – 97.17) | -3.40 (-3.64 – -3.16) |
| Guizhou | 255.14 (201.34 – 289.65) | 118.62 (96.56 – 139.81) | -2.62 (-2.79 – -2.44) |
| Hainan | 145.94 (111.91 – 197.29) | 75.81 (60.49 – 92.99) | -2.18 (-2.42 – -1.94) |
| Hebei | 142.78 (119.85 – 169.84) | 55.09 (41.52 – 90.75) | -3.97 (-4.24 – -3.69) |
| Heilongjiang | 187.00 (160.34 – 213.88) | 46.20 (36.01 – 86.79) | -5.38 (-5.88 – -4.87) |
| Henan | 152.73 (130.36 – 177.37) | 45.77 (35.13 – 80.26) | -4.34 (-4.76 – -3.92) |
| Hong Kong * | 55.27 (49.90 – 69.60) | 20.14 (13.69 – 38.47) | -3.70 (-3.96 – -3.44) |
| Hubei | 163.12 (139.53 – 181.46) | 68.84 (55.92 – 85.03) | -3.29 (-3.49 – -3.09) |
| Hunan | 248.25 (167.36 – 288.37) | 78.49 (65.00 – 93.35) | -4.57 (-4.94 – -4.19) |
| Inner Mongolia | 209.23 (173.66 – 237.47) | 68.15 (55.85 – 89.91) | -4.21 (-4.42 – -3.99) |
| Jiangsu | 245.08 (150.14 – 279.54) | 52.86 (42.93 – 66.53) | -6.05 (-6.36 – -5.74) |
| Jiangxi | 291.95 (190.41 – 338.16) | 85.23 (69.52 – 99.59) | -4.44 (-4.56 – -4.32) |
| Jilin | 143.38 (127.25 – 189.61) | 30.47 (23.39 – 74.84) | -5.75 (-6.12 – -5.38) |
| Liaoning | 116.81 (95.76 – 147.50) | 31.98 (23.75 – 68.47) | -5.01 (-5.54 – -4.47) |
| Macao * | 96.72 (70.70 – 111.80) | 42.97 (29.80 – 55.67) | -2.97 (-3.09 – -2.85) |
| Ningxia | 225.97 (182.08 – 260.81) | 76.52 (60.84 – 102.31) | -3.82 (-3.95 – -3.69) |
| Qinghai | 322.20 (227.53 – 376.09) | 165.92 (134.29 – 189.82) | -2.39 (-2.48 – -2.29) |
| Shaanxi | 226.02 (184.13 – 259.75) | 52.83 (40.07 – 89.18) | -5.53 (-5.87 – -5.19) |
| Shandong | 229.49 (161.51 – 271.85) | 46.86 (37.36 – 68.39) | -5.99 (-6.17 – -5.81) |
| Shanghai | 165.27 (113.84 – 192.12) | 35.26 (27.31 – 47.70) | -5.92 (-6.15 – -5.69) |
| Shanxi | 204.01 (173.76 – 231.60) | 56.42 (42.62 – 97.83) | -4.74 (-4.87 – -4.61) |
| Sichuan | 295.51 (185.77 – 337.58) | 137.61 (102.20 – 162.71) | -2.87 (-3.06 – -2.68) |
| Tianjin | 147.60 (122.80 – 166.78) | 27.76 (20.72 – 52.87) | -6.56 (-6.88 – -6.23) |
| Tibet | 345.07 (270.93 – 488.87) | 181.44 (146.11 – 215.83) | -3.00 (-3.73 – -2.27) |
| Xinjiang | 288.25 (200.47 – 342.10) | 146.73 (108.68 – 180.78) | -2.76 (-3.04 – -2.48) |
| Yunnan | 316.21 (217.52 – 364.25) | 145.39 (121.38 – 165.63) | -2.66 (-2.76 – -2.56) |
| Zhejiang | 243.44 (150.31 – 283.97) | 45.68 (36.34 – 58.49) | -6.34 (-6.72 – -5.97) |

* Special Administrative Region of China. ASR, age-standardized rate; EAPC, estimated annual percentage change.
