# Supplementary material for: The Burden of COPD in China and Its Provinces: Findings From the Global Burden of Disease Study 2019
Source: Front Public Health. 2022 Jun 3;10:859499. doi: 10.3389/fpubh.2022.859499 (PMC9215345; doi:10.3389/fpubh.2022.859499)
Supplement: Supplementary file 3 [file Data_Sheet_1.zip › Table 5.DOCX]

**Supplementary Table 5. The age-standardized disability-adjusted life years rates of COPD in 1990 and 2019, and their temporal trends from 1990 to 2019 at provincial level of China.**

| Province | ASR in 1990 (per 100,000) | ASR in 2019 (per 100,000) | EAPC (1990-2019) |
| --- | --- | --- | --- |
| Anhui | 3630.79 (2787.08 – 4233.92) | 902.08 (746.07 – 1240.71) | -5.23 (-5.45 – -5.01) |
| Beijing | 1907.91 (1656.22 – 2119.57) | 427.65 (345.77 – 627.78) | -5.98 (-6.30 – -5.65) |
| Chongqing | 5878.57 (3192.55 – 7059.53) | 1875.81 (1450.34 – 2233.56) | -4.06 (-4.14 – -3.98) |
| Fujian | 3747.95 (2695.41 – 4282.15) | 779.22 (650.17 – 1011.78) | -5.96 (-6.27 – -5.64) |
| Gansu | 5196.39 (3544.91 – 5989.44) | 1880.39 (1602.46 – 2160.46) | -3.52 (-3.70 – -3.35) |
| Guangdong | 3558.63 (2492.85 – 4091.06) | 834.39 (719.98 – 982.80) | -5.46 (-5.87 – -5.05) |
| Guangxi | 3507.14 (2640.38 – 4002.46) | 1263.08 (1054.60 – 1510.64) | -3.55 (-3.72 – -3.37) |
| Guizhou | 4380.99 (3430.74 – 5067.89) | 1911.29 (1581.28 – 2234.59) | -2.87 (-3.03 – -2.70) |
| Hainan | 2586.96 (2026.12 – 3380.94) | 1201.95 (988.37 – 1437.04) | -2.69 (-2.88 – -2.51) |
| Hebei | 2295.71 (1938.30 – 2684.87) | 944.45 (767.27 – 1406.69) | -3.64 (-3.88 – -3.40) |
| Heilongjiang | 3043.49 (2624.40 – 3478.20) | 840.49 (694.29 – 1394.59) | -4.90 (-5.33 – -4.46) |
| Henan | 2503.22 (2172.64 – 2858.70) | 831.31 (682.35 – 1289.16) | -3.98 (-4.32 – -3.63) |
| Hong Kong * | 1130.88 (1052.71 – 1297.69) | 438.07 (348.49 – 666.04) | -3.53 (-3.73 – -3.34) |
| Hubei | 2748.62 (2377.21 – 3052.63) | 1136.74 (960.55 – 1368.01) | -3.33 (-3.47 – -3.18) |
| Hunan | 4021.16 (2755.58 – 4694.58) | 1341.06 (1141.84 – 1561.59) | -4.27 (-4.56 – -3.99) |
| Inner Mongolia | 3432.61 (2902.33 – 3920.53) | 1119.60 (953.80 – 1434.91) | -4.20 (-4.39 – -4.00) |
| Jiangsu | 3685.09 (2420.48 – 4173.50) | 865.97 (736.13 – 1042.58) | -5.68 (-5.95 – -5.41) |
| Jiangxi | 4923.84 (3212.12 – 5707.53) | 1341.44 (1133.14 – 1536.33) | -4.72 (-4.85 – -4.60) |
| Jilin | 2366.38 (2106.88 – 3067.25) | 568.71 (462.63 – 1149.00) | -5.30 (-5.63 – -4.97) |
| Liaoning | 1887.63 (1589.01 – 2296.31) | 592.86 (477.58 – 1058.75) | -4.46 (-4.92 – -4.01) |
| Macao * | 1756.07 (1376.66 – 1993.28) | 774.32 (605.15 – 947.56) | -2.99 (-3.08 – -2.91) |
| Ningxia | 3674.67 (2970.46 – 4269.40) | 1220.95 (994.50 – 1559.29) | -3.93 (-4.03 – -3.83) |
| Qinghai | 5547.59 (3879.84 – 6541.77) | 2693.12 (2195.82 – 3095.03) | -2.62 (-2.74 – -2.49) |
| Shaanxi | 3664.05 (3007.53 – 4268.48) | 893.82 (714.92 – 1362.87) | -5.36 (-5.65 – -5.07) |
| Shandong | 3684.13 (2641.10 – 4297.38) | 849.88 (717.60 – 1142.71) | -5.49 (-5.63 – -5.35) |
| Shanghai | 2568.09 (1838.87 – 2989.19) | 563.17 (463.93 – 719.74) | -5.85 (-6.09 – -5.62) |
| Shanxi | 3243.38 (2781.10 – 3688.41) | 920.01 (725.83 – 1471.00) | -4.66 (-4.79 – -4.54) |
| Sichuan | 5251.32 (3180.46 – 6041.51) | 2201.07 (1684.91 – 2595.79) | -3.31 (-3.51 – -3.10) |
| Tianjin | 2301.11 (1958.02 – 2555.03) | 521.21 (421.91 – 837.39) | -5.80 (-6.06 – -5.53) |
| Tibet | 6238.23 (4878.81 – 8646.27) | 2943.41 (2397.16 – 3479.53) | -3.39 (-4.08 – -2.70) |
| Xinjiang | 4897.95 (3360.65 – 5879.56) | 2337.46 (1785.78 – 2825.39) | -2.91 (-3.18 – -2.64) |
| Yunnan | 5440.77 (3622.35 – 6381.81) | 2306.54 (1949.53 – 2625.29) | -2.98 (-3.09 – -2.87) |
| Zhejiang | 3792.34 (2424.61 – 4419.10) | 737.69 (619.01 – 913.74) | -6.18 (-6.51 – -5.86) |

* Special Administrative Region of China. ASR, age-standardized rate; EAPC, estimated annual percentage change.
