# Supplementary material for: The Burden of COPD in China and Its Provinces: Findings From the Global Burden of Disease Study 2019
Source: Front Public Health. 2022 Jun 3;10:859499. doi: 10.3389/fpubh.2022.859499 (PMC9215345; doi:10.3389/fpubh.2022.859499)
Supplement: Supplementary file 3 [file Data_Sheet_1.zip › Table 6.DOCX]

**Supplementary Table 6. The age-standardized years lived with disability rates of COPD in 1990 and 2019, and their temporal trends from 1990 to 2019 at provincial level of China.**

| Province | ASR in 1990 (per 100,000) | ASR in 2019 (per 100,000) | EAPC (1990-2019) |
| --- | --- | --- | --- |
| Anhui | 338.69 (279.91 ‒ 391.69) | 228.42 (187.13 ‒ 267.18) | -1.60 (-1.81 – -1.38) |
| Beijing | 280.97 (229.27 ‒ 328.73) | 140.22 (113.00 ‒ 165.34) | -2.79 (-3.04 – -2.54) |
| Chongqing | 365.02 (304.37 ‒ 417.48) | 316.01 (259.27 ‒ 367.12) | -0.59 (-0.76 – -0.43) |
| Fujian | 338.66 (280.59 ‒ 392.33) | 205.78 (168.45 ‒ 241.78) | -1.99 (-2.19 – -1.79) |
| Gansu | 340.83 (284.80 ‒ 388.91) | 299.89 (248.52 ‒ 346.95) | -0.51 (-0.67 – -0.35) |
| Guangdong | 339.57 (278.69 ‒ 394.76) | 242.14 (198.73 ‒ 282.81) | -1.32 (-1.49 – -1.14) |
| Guangxi | 323.69 (265.63 ‒ 375.09) | 242.23 (197.23 ‒ 281.39) | -1.13 (-1.28 – -0.98) |
| Guizhou | 328.30 (273.17 ‒ 379.66) | 288.16 (236.33 ‒ 332.86) | -0.55 (-0.70 – -0.40) |
| Hainan | 305.82 (249.25 ‒ 358.38) | 205.35 (168.88 ‒ 241.13) | -1.59 (-1.73 – -1.44) |
| Hebei | 265.34 (216.27 ‒ 311.29) | 209.01 (171.02 ‒ 245.79) | -0.92 (-1.02 – -0.81) |
| Heilongjiang | 333.03 (278.08 ‒ 383.96) | 223.19 (181.97 ‒ 260.44) | -1.57 (-1.83 – -1.31) |
| Henan | 313.00 (256.54 ‒ 363.57) | 233.31 (191.35 ‒ 271.84) | -1.15 (-1.33 – -0.97) |
| Hong Kong * | 252.13 (207.33 ‒ 293.74) | 166.07 (136.19 ‒ 194.93) | -1.79 (-1.99 – -1.59) |
| Hubei | 326.67 (267.25 ‒ 377.93) | 259.64 (212.32 ‒ 302.05) | -0.91 (-1.07 – -0.76) |
| Hunan | 337.70 (279.92 ‒ 385.56) | 279.54 (230.13 ‒ 324.36) | -0.72 (-0.91 – -0.54) |
| Inner Mongolia | 333.40 (279.63 ‒ 383.50) | 244.67 (200.84 ‒ 284.85) | -1.23 (-1.46 – -1.00) |
| Jiangsu | 355.63 (294.15 ‒ 410.60) | 240.03 (196.41 ‒ 280.33) | -1.53 (-1.72 – -1.35) |
| Jiangxi | 345.06 (287.35 ‒ 396.01) | 257.75 (211.66 ‒ 299.64) | -1.17 (-1.37 – -0.98) |
| Jilin | 268.40 (218.45 ‒ 313.39) | 162.69 (130.81 ‒ 193.03) | -2.05 (-2.32 – -1.77) |
| Liaoning | 291.84 (238.86 ‒ 339.01) | 181.96 (149.23 ‒ 214.98) | -1.84 (-2.06 – -1.62) |
| Macao * | 314.98 (257.19 ‒ 366.27) | 212.83 (174.93 ‒ 247.44) | -1.63 (-1.81 – -1.45) |
| Ningxia | 314.23 (259.97 ‒ 363.57) | 231.83 (190.13 ‒ 269.83) | -1.26 (-1.45 – -1.06) |
| Qinghai | 334.37 (277.31 ‒ 383.16) | 296.86 (242.89 ‒ 344.80) | -0.44 (-0.62 – -0.26) |
| Shaanxi | 267.04 (218.96 ‒ 311.55) | 193.42 (157.90 ‒ 226.62) | -1.28 (-1.42 – -1.13) |
| Shandong | 366.43 (301.20 ‒ 421.24) | 250.06 (205.07 ‒ 293.07) | -1.50 (-1.71 – -1.28) |
| Shanghai | 315.46 (258.62 ‒ 365.85) | 158.44 (129.58 ‒ 186.95) | -2.75 (-3.06 – -2.44) |
| Shanxi | 307.16 (253.41 ‒ 358.33) | 184.53 (150.44 ‒ 217.65) | -2.03 (-2.26 – -1.81) |
| Sichuan | 359.08 (299.89 ‒ 407.23) | 328.33 (267.41 ‒ 379.64) | -0.28 (-0.43 – -0.13) |
| Tianjin | 314.70 (258.62 ‒ 366.84) | 174.90 (142.20 ‒ 205.98) | -2.39 (-2.62 – -2.16) |
| Tibet | 323.96 (269.56 ‒ 371.01) | 256.93 (209.56 ‒ 299.61) | -0.96 (-1.06 – -0.87) |
| Xinjiang | 318.18 (264.51 ‒ 365.37) | 299.21 (245.14 ‒ 345.94) | -0.23 (-0.40 – -0.06) |
| Yunnan | 336.87 (279.41 ‒ 382.85) | 300.00 (246.86 ‒ 349.41) | -0.45 (-0.59 – -0.30) |
| Zhejiang | 356.10 (293.20 ‒ 407.11) | 211.47 (172.95 ‒ 247.81) | -2.06 (-2.34 – -1.78) |

* Special Administrative Region of China. ASR, age-standardized rate; EAPC, estimated annual percentage change.
