# Supplementary material for: The Burden of COPD in China and Its Provinces: Findings From the Global Burden of Disease Study 2019
Source: Front Public Health. 2022 Jun 3;10:859499. doi: 10.3389/fpubh.2022.859499 (PMC9215345; doi:10.3389/fpubh.2022.859499)
Supplement: Supplementary file 3 [file Data_Sheet_1.zip › Table 7.DOCX]

**Supplementary Table 7. The age-standardized years of life lost rates of COPD in 1990 and 2019, and their temporal trends from 1990 to 2019 at provincial level of China.**

| Province | ASR in 1990 (per 100,000) | ASR in 2019 (per 100,000) | EAPC (1990-2019) |
| --- | --- | --- | --- |
| Anhui | 3292.10 (2455.31 ‒ 3849.89) | 673.66 (528.09 ‒ 1005.69) | -5.90 (-6.16 ‒ -5.64) |
| Beijing | 1626.94 (1378.54 ‒ 1838.37) | 287.43 (214.23 ‒ 485.70) | -6.93 (-7.31 ‒ -6.54) |
| Chongqing | 5513.55 (2826.02 ‒ 6681.69) | 1559.80 (1132.22 ‒ 1898.10) | -4.47 (-4.56 ‒ -4.38) |
| Fujian | 3409.29 (2378.94 ‒ 3936.52) | 573.44 (453.86 ‒ 803.13) | -6.71 (-7.08 ‒ -6.33) |
| Gansu | 4855.57 (3177.58 ‒ 5662.82) | 1580.49 (1308.17 ‒ 1858.07) | -3.87 (-4.07 ‒ -3.67) |
| Guangdong | 3219.07 (2131.91 ‒ 3746.62) | 592.25 (489.82 ‒ 724.90) | -6.33 (-6.84 ‒ -5.83) |
| Guangxi | 3183.45 (2328.64 ‒ 3673.45) | 1020.85 (815.80 ‒ 1266.13) | -3.92 (-4.13 ‒ -3.72) |
| Guizhou | 4052.69 (3123.96 ‒ 4732.25) | 1623.13 (1303.73 ‒ 1948.12) | -3.15 (-3.33 ‒ -2.96) |
| Hainan | 2281.14 (1731.30 ‒ 3083.54) | 996.60 (789.98 ‒ 1229.74) | -2.88 (-3.09 ‒ -2.67) |
| Hebei | 2030.37 (1679.37 ‒ 2410.71) | 735.44 (562.17 ‒ 1205.55) | -4.18 (-4.46 ‒ -3.89) |
| Heilongjiang | 2710.47 (2293.56 ‒ 3146.99) | 617.31 (480.12 ‒ 1162.26) | -5.61 (-6.11 ‒ -5.11) |
| Henan | 2190.22 (1865.10 ‒ 2549.63) | 598.00 (456.82 ‒ 1045.62) | -4.64 (-5.06 ‒ -4.23) |
| Hong Kong * | 878.75 (810.59 ‒ 1043.94) | 272.00 (189.30 ‒ 497.09) | -4.29 (-4.57 ‒ -4.01) |
| Hubei | 2421.95 (2066.52 ‒ 2716.07) | 877.10 (707.82 ‒ 1106.30) | -3.80 (-3.97 ‒ -3.64) |
| Hunan | 3683.46 (2431.89 ‒ 4353.87) | 1061.51 (872.26 ‒ 1281.36) | -4.83 (-5.15 ‒ -4.50) |
| Inner Mongolia | 3099.21 (2565.04 ‒ 3591.20) | 874.93 (710.14 ‒ 1189.40) | -4.72 (-4.93 ‒ -4.50) |
| Jiangsu | 3329.46 (2039.99 ‒ 3815.25) | 625.94 (504.83 ‒ 788.13) | -6.54 (-6.86 ‒ -6.22) |
| Jiangxi | 4578.78 (2875.02 ‒ 5367.88) | 1083.68 (881.86 ‒ 1270.51) | -5.22 (-5.35 ‒ -5.08) |
| Jilin | 2097.98 (1859.78 ‒ 2817.48) | 406.02 (311.57 ‒ 980.12) | -6.06 (-6.45 ‒ -5.67) |
| Liaoning | 1595.79 (1306.30 ‒ 2022.91) | 410.90 (302.52 ‒ 878.35) | -5.20 (-5.75 ‒ -4.64) |
| Macao * | 1441.09 (1061.36 ‒ 1678.92) | 561.49 (394.44 ‒ 733.71) | -3.40 (-3.52 ‒ -3.29) |
| Ningxia | 3360.44 (2645.71 ‒ 3963.45) | 989.12 (779.72 ‒ 1330.25) | -4.33 (-4.44 ‒ -4.22) |
| Qinghai | 5213.22 (3531.56 ‒ 6195.36) | 2396.26 (1899.96 ‒ 2794.91) | -2.81 (-2.94 ‒ -2.68) |
| Shaanxi | 3397.00 (2724.69 ‒ 3980.19) | 700.39 (526.05 ‒ 1182.02) | -5.98 (-6.32 ‒ -5.63) |
| Shandong | 3317.70 (2277.16 ‒ 3922.01) | 599.82 (476.18 ‒ 880.14) | -6.38 (-6.55 ‒ -6.20) |
| Shanghai | 2252.63 (1520.69 ‒ 2667.53) | 404.73 (313.29 ‒ 560.02) | -6.60 (-6.85 ‒ -6.34) |
| Shanxi | 2936.22 (2483.02 ‒ 3371.73) | 735.48 (545.34 ‒ 1292.04) | -5.10 (-5.22 ‒ -4.97) |
| Sichuan | 4892.24 (2817.21 ‒ 5690.38) | 1872.75 (1366.56 ‒ 2271.52) | -3.65 (-3.88 ‒ -3.43) |
| Tianjin | 1986.41 (1647.58 ‒ 2237.63) | 346.32 (257.26 ‒ 670.76) | -6.76 (-7.07 ‒ -6.45) |
| Tibet | 5914.27 (4578.53 ‒ 8334.24) | 2686.49 (2137.57 ‒ 3230.26) | -3.60 (-4.36 ‒ -2.83) |
| Xinjiang | 4579.77 (3033.65 ‒ 5558.65) | 2038.25 (1478.20 ‒ 2514.74) | -3.19 (-3.48 ‒ -2.90) |
| Yunnan | 5103.90 (3276.58 ‒ 6029.53) | 2006.54 (1662.69 ‒ 2329.32) | -3.24 (-3.36 ‒ -3.12) |
| Zhejiang | 3436.24 (2058.46 ‒ 4042.21) | 526.22 (417.11 ‒ 694.68) | -7.07 (-7.46 ‒ -6.68) |

* Special Administrative Region of China. ASR, age-standardized rate; EAPC, estimated annual percentage change.
